# Functional Agents to Biologically Control Deoxynivalenol Contamination in Cereal Grains

**DOI:** 10.3389/fmicb.2016.00395

**Published:** 2016-03-30

**Authors:** Ye Tian, Yanglan Tan, Na Liu, Yucai Liao, Changpo Sun, Shuangxia Wang, Aibo Wu

**Affiliations:** ^1^SIBS-UGENT-SJTU Joint Laboratory of Mycotoxin Research, Key Laboratory of Food Safety Research, Institute for Nutritional Sciences, Shanghai Institutes for Biological Sciences, Chinese Academy of SciencesShanghai, China; ^2^College of Plant Science and Technology, Huazhong Agricultural UniversityWuhan, China; ^3^Academy of State Administration of GrainBeijing, China

**Keywords:** mycotoxin, deoxynivalenol (DON), *Fusarium*, biological agents, control

## Abstract

Mycotoxins, as microbial secondary metabolites, frequently contaminate cereal grains and pose a serious threat to human and animal health around the globe. Deoxynivalenol (DON), a commonly detected *Fusarium* mycotoxin, has drawn utmost attention due to high exposure levels and contamination frequency in the food chain. Biological control is emerging as a promising technology for the management of DON contamination. Functional biological control agents (BCAs), which include antagonistic microbes, natural fungicides derived from plants and detoxification enzymes, can be used to control DON contamination at different stages of grain production. In this review, studies regarding different biological agents for DON control in recent years are summarized for the first time. Furthermore, this article highlights the significance of BCAs for controlling DON contamination, as well as the need for more practical and efficient BCAs concerning food safety.

## Introduction

**FIGURE 1:**
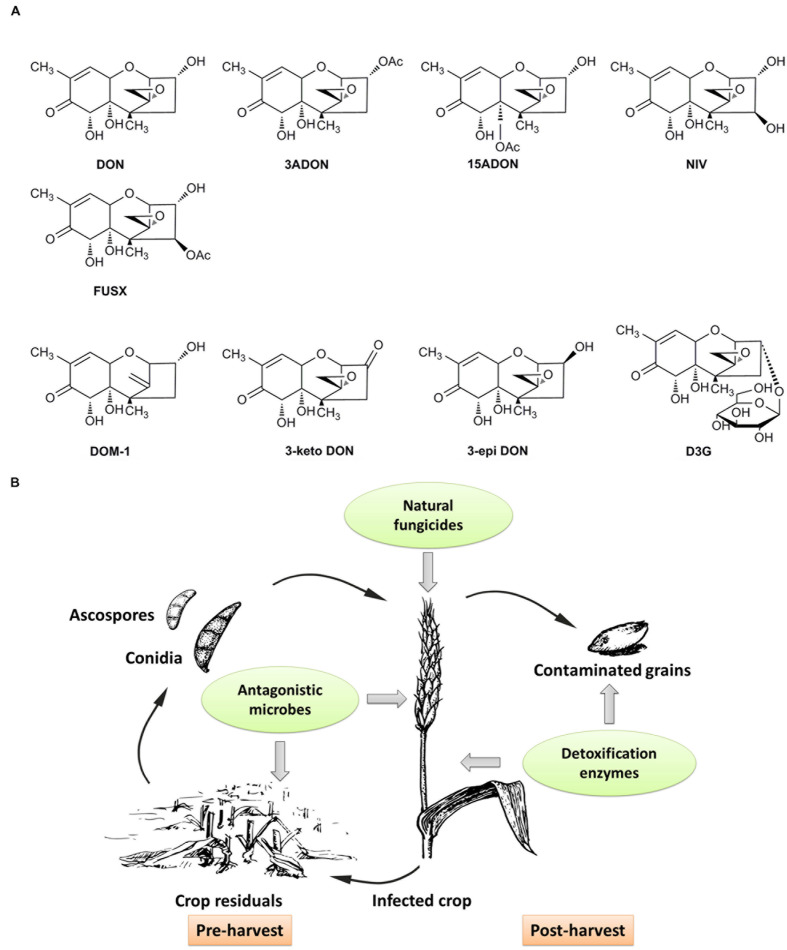
**Chemical structures of major type B trichothecenes and the detoxification products of DON, and a schematic of BCAs on control of DON contamination at different stages of grain production.** **(A)** Major B type trichothecenes include DON, 3ADON, 15ADON, NIV and FUSX. Detoxification products of DON mainly include DOM-1, 3-keto DON, 3-epi DON and D3G. **(B)** Antagonistic microbes can be applied to crop residuals to inhibit sporulation, or to spikes with natural fungicides to inhibit the growth and DON production of pathogens. Contaminated grains can be treated with enzymes to detoxification after harvest, and the enzymes also can be expressed in genetically modified crops to detoxify DON and increase crop resistance to pathogens.

Mycotoxins are secondary metabolites produced by fungi and may exert toxic effects on plants, animals and humans (Goswami and Kistler, 2004; Yu and Keller, 2005). Trichothecenes, a group of sesquiterpenoid mycotoxins, are commonly found in grains worldwide. B type trichothecenes that are common contaminants of grains, are characterized by a keto functional group at C-8 in their molecular structures (Richard, 2007; McCormick et al., 2011; Mohamed Anwar et al., 2014). Some common B type trichothecenes include deoxynivalenol (DON), 3-acetyldeoxynivalenol (3ADON), 15-acetyldeoxynivalenol (15ADON), nivalenol (NIV) and fusarenon X (FUSX) (**Figure 1A**) (Arunachalam and Doohan, 2013). Among them, DON, also known as vomitoxin, is the most frequently detected and economically important mycotoxin in cereal grains. DON contamination in cereal grains is a global problem. DON is predominantly produced by *Fusarium graminearum* and *Fusarium culmorum*. These phytopathogens can infect crops in the field and cause a destructive disease called *Fusarium* head blight (FHB) or scab (McMullen et al., 2012). As a virulence factor of these phytopathogens, mycotoxin DON facilitates the spread of *Fusarium* strains within infected tissue and contributes to the symptoms of FHB disease (Bai et al., 2002).

FHB caused by *Fusarium* strains can lead to enormous losses of yield and quality in cereal grains. Moreover, DON contamination poses a great threat to public health and food safety (Wagacha and Muthomi, 2008). Consumption of DON-contaminated food can cause serious gastroenteritis including diarrhea, nausea, vomition, and abdominal pain in humans (Pestka, 2010; Sobrova et al., 2010; Pinton et al., 2012; da Rocha et al., 2014). Thus, DON contamination control is a crucial issue for mitigating economic losses and improving food safety in the food chain. Currently, some effective measures including crop rotation, selection of resistant wheat lines, fungicides application and biological control agents (BCAs) have been put into action to control DON contamination in grain production (Dill-Macky and Jones, 2000; Edwards and Godley, 2010; Wegulo et al., 2015). Of the measures mentioned above, application of synthetic fungicides is relatively successful for control of these phytopathogens and mycotoxin production. However, synthetic fungicides are not economical for long-term use, as well as causing a series of undesirable effects on the environment (Mesterhazy et al., 2011; Schoneberg et al., 2015). Biological control of DON contamination is emerging as a green approach. Functional BCAs include antagonistic microbes, natural fungicides derived from plants which inhibit the development and mycotoxin production, and enzymes from beneficial organisms for DON detoxification after production. Until now, several reviews on managing FHB or mycotoxins detoxification with different strategies have been published (Yuen and Schoneweis, 2007; Awad et al., 2010; He et al., 2010; Karlovsky, 2011; McCormick, 2013; Wegulo et al., 2015). Here we are focusing on recent progresses in various BCAs to achieve DON contamination control (**Table 1**), which will be reviewed briefly in the next section. This will be beneficial to understand the exploration and application of BCAs in the field of DON contamination control.

**Table 1:** Functional BCAs of controlling DON contamination mentioned in this review.

	Functional BCAs	Origin	Mechanisms of controlling DON contamination	Reference
Antagonistic microbes	*Trichoderma* strains	Isolated from soil or plants	Inhibiting sporulation, growth and/or mycotoxin DON production of pathogens	Schoneberg et al., 2015


	*Trichoderma* strains	Isolated from soil or plants		Matarese et al., 2012
	*Trichoderma atroviride* P1	Isolated from soil		Lutz et al., 2003
	*Clonostachys rosea*	Isolated from cereal crops		Schoneberg et al., 2015
	*Cladosporium cladosporioides*	Isolated from cereal crops		Schoneberg et al., 2015
	*Aureobasidium pullulans*	Isolated from winter wheat grains		Wachowska and Głowacka, 2014
	*Cryptococcus* strains	Unknow		Schisler et al., 2011
	*Pseudomonas* strains	Isolated from infected spikelets		Yoshida et al., 2012
	*Bacillus subtilis* SG6	Isolated from wheat anthers		Zhao et al., 2014
	*Bacillus subtilis* RC 218 and *Brevibacillus* sp. RC 263	Isolated from wheat anthers		Palazzini et al., 2016
	*Bacillus amyloliquefaciens*	Isolated from peanut shells		Shi et al., 2014
	*Shewanella algae* strain YM8	Isolated from sea sediment		Gong et al., 2015

Natural fungicides	Phenolic compounds	Extracts of *Spirulina* strains	Inhibiting growth and/or mycotoxin DON production of pathogens	Pagnussatt et al., 2013
	Phenolic compounds	Extracts of *Spirulina* strains		Pagnussatt et al., 2014
	Phenolic acids	Extracts of maizes		Gauthier et al., 2016
	Essential oils	Extracts of cinnamon, clove, lemongrass, oregano and palmarosa		Marín et al., 2004
				Velluti et al., 2004
	Essential oils	Extracts of *Ocimum sanctum*		Kalagatur et al., 2015

Detoxification enzymes	Unknown enzyme	*Bacillus licheniformis* and *Bacillus subtilis* provided by Jiangxi-OAI Joint Research Institute, Nanchang University, China	Detoxifying DON to less toxic products	Cheng et al., 2010
	Unknown enzyme	A strain of *Aspergillus* NJA1 isolated from soil		He et al., 2008
	Deepoxidase	*Bacillus* sp. LS100 isolated from chicken digesta		Li et al., 2011
	Deepoxidase	Bacteria isolated from intestines of chicken		Young et al., 2007
	Deepoxidase	A strain of *Bacillus* isolated from intestinal track of fish		Guan et al., 2009
	Deepoxidase	A mixed microbial culture including six bacterial genera found from soil		Islam et al., 2012
	Deepoxidase	Fecal microbiota isolated from intestines of human		Gratz et al., 2013
	Oxidase and epimerase	*Nocardioides* sp. strain WSN05-2 isolated from a wheat field		Ikunaga et al., 2011
	Oxidase and epimerase	Genus of *Nocardioides* and *Devosia* isolated from field soils and wheat leaves		Sato et al., 2012
	Oxidase and epimerase	*Devosia mutans* 17-2-E-8 isolated from an agricultural soil		He et al., 2015
	UDP-glucosyltransferase	*Arabidopsis thaliana*		Poppenberger et al., 2003
	UDP-glucosyltransferase	*Triticum aestivum L. cv. Wangshuibai*		Lulin et al., 2010
	UDP-glucosyltransferase	Barley		Schweiger et al., 2010
	UDP-glucosyltransferase	*Arabidopsis thaliana*		Shin et al., 2012
	UDP-glucosyltransferase	Barley		Li et al., 2015


## Functional BCAs Against DON Contamination

### Antagonistic Microbes

Previous results have demonstrated the positive linear relationship between the occurrence of FHB and DON contamination (Wegulo et al., 2011; Hernandez Nopsa et al., 2012; Wegulo, 2012). Therefore, managing FHB plays a key role in controlling DON contamination (Yuen and Schoneweis, 2007). Selection of available antagonistic microbes that suppress mycelia growth, sporulation and mycotoxin production of pathogens is indispensable for the purpose of managing FHB (Pfliegler et al., 2015), of which antagonistic fungi and bacteria have gained significant attention in the past.

Major fungal antagonists comprise *Trichoderma* strains, *Clonostachys rosea*, *Cladosporium cladosporioides* (Schoneberg et al., 2015), *Aureobasidium pullulans* (Wachowska and Głowacka, 2014) and *Cryptococcus* strains (Schisler et al., 2011). *Trichoderma* strains have been widely investigated, because they grow fast as competitors to significantly reduce the colony areas of *Fusarium* strains, and inhibit the pathogen spread by antibiotic production (Matarese et al., 2012; Schoneberg et al., 2015). Another important control mechanism is mycoparasitism mediated by production of cell wall degrading enzymes including cellulases, chitinase and glucanases (Vinale et al., 2008; Mukherjee et al., 2013). On the other hand, during competition between *Fusarium* and *Trichoderma*, DON production, as a negative signal against antagonism, can repress one chitinase gene (nag1) expression in a *Trichoderma atroviride* strain P1 (Lutz et al., 2003).

Bacterial strains in the genus of *Bacillus* and *Pseudomonas* have also been widely explored as potential BCAs against FHB in recent years (Yoshida et al., 2012). Most antagonistic bacterial strains belong to endophytic microbes inhabiting plant or rhizosphere without leading to diseases or adverse effects (Dal Bello et al., 2002). *Bacillus subtilis* SG6 isolated from wheat anthers showed a remarkable inhibitory effect on mycelial growth, sporulation and DON production of *F. graminearum* (Zhao et al., 2014). Moreover, *B. subtilis* RC 218 and *Brevibacillus* sp. RC 263 isolated from wheat anthers could effectively reduce the incidence and severity of FHB and DON accumulation under semi controlled field conditions (Palazzini et al., 2016). In another study, bacterial strains isolated from peanut shells exhibited potent inhibition to the growth and DON production of *F. graminearum*, and the tested strains with the strongest inhibitory effect were identified as *B. amyloliquefaciens* (Shi et al., 2014). Besides, a *Shewanella algae* strain YM8 isolated from sea sediment, which can produce volatile organic compounds, has a broad spectrum of inhibition activity against nine agronomically important phytopathogens including *F. graminearum* (Gong et al., 2015). This research indicates that marine bacteria can be a potential source for effective agents to control the growth and mycotoxin production of pathogens in the field and during storage.

The above mentioned antagonistic microbes can be applied to crop residuals to inhibit ascospores and conidia production, or directly used on spikes to restrict the development and mycotoxin production of pathogens (**Figure 1B**) (Xue et al., 2014; Schoneberg et al., 2015; Wegulo et al., 2015).

### Natural Fungicides

In order to decrease the use of synthetic fungicides, a green alternative strategy with natural fungicides can be used to inhibit pathogens (da Cruz Cabral et al., 2013). The restriction on applications of chemical fungicides has increased the demand of natural fungicides (Terzi et al., 2014). As potential sources of natural fungicides, metabolites from plants, including phenolic compounds and essential oils, have been researched for activities that inhibit pathogen development and mycotoxin production in recent years (Esper et al., 2014; Pagnussatt et al., 2014).

Phenolic compounds derived from *Spirulina* strains exerted efficient antifungal activity against *F. graminearum* (Pagnussatt et al., 2013, 2014). Moreover, a recent work has indicated that chlorogenic acid, a common phenolic acid, can be transformed by *F. graminearum*, generating some forms of metabolites which are even more efficient in limiting mycelial growth and DON production (Gauthier et al., 2016). This study provides a new understanding on the role of phenolic compounds in their antifungal activities.

Essential oils extracted from plants usually contain some antimicrobial or antioxidant compounds, and they are regarded as good choices of natural fungicides (Bakkali et al., 2008). For instance, essential oils extracted from cinnamon, clove, oregano, palmarosa and lemongrass were selected to test their anti-mycotoxigenic activity. All these essential oils could prevent DON accumulation in *F. graminearum*-infected grains, and the clove essential oil was the most effective (Marín et al., 2004). In addition, another study found that environmental factors, such as water activity and temperature, could influence the anti-mycotoxigenic activity of essential oils (Velluti et al., 2004). Recent research shows that *Ocimum sanctum* essential oil has a prominent antagonistic activity on the growth of *F. graminearum* (Kalagatur et al., 2015). All these studies show that natural fungicides that are sourced from functional plant metabolites have great potentials in controlling DON contamination.

### Detoxification Enzymes

As complementary to management of the incidence and severity of FHB by antagonistic microbes and natural fungicides, detoxification of DON in contaminated grains could also reduce food safety risk and economic losses effectively (Awad et al., 2010; Jard et al., 2011; Karlovsky, 2011; McCormick, 2013). A number of conventional physical and chemical approaches have been used to remove DON from contaminated grains, but the loss of nutritional value or potential safety problems should not be ignored (He et al., 2010). Therefore, detoxifying DON by enzymatic reactions can be an attractive approach for controlling DON contamination.

Enzymatic reactions of DON detoxification may include deepoxidation, oxidation, epimerization and glycosylation. The detoxification products from these reactions, such as DOM-1, 3-keto DON, 3-epi DON and DON-3-glucoside (D3G), are shown in **Figure 1A**. In addition, some studies with unknown detoxification products were also reported. For example, *B. licheniformis* and *B. subtilis* strains were proved to degrade DON under anaerobic conditions, but the detoxification products in this transformation remained unknown (Cheng et al., 2010). A strain of *Aspergillus* (NJA1) isolated from soil could convert DON to an unknown product with a molecule weight of 18.1 kDa (He et al., 2008). In another study, strains of *Rhizopus oryzae* and *Aspergillus oryzae* can degrade DON in submerged fermentation, but the degradation mechanism was mainly explained by toxin absorption (Garda-Buffon and Badiale-Furlong, 2010). Therefore, deepoxidation, oxidation, epimerization and glycosylation are available enzymatic detoxification processes.

#### Deepoxidation

The active epoxide group in DON determines its toxicity for interrupting protein synthesis. DON can be deepoxidated to deepoxy DON (DOM-1) which is much less toxic. This process exhibits a promising detoxification approach in contaminated grains (Karlovsky, 2011; Li et al., 2011). Bacteria from the intestines of chicken could convert DON to DOM-1 under oxygen free conditions (Young et al., 2007). In aerobic conditions, a *Bacillus* strain isolated from intestinal track of fish can also deepoxidate DON in contaminated corn (Guan et al., 2009). A mixed microbial culture including six bacterial genera found in soil was capable of converting DON to DOM-1 under aerobic conditions with a higher transformation efficiency compared to anaerobic conditions (Islam et al., 2012). Interestingly, the human fecal microbiota from one volunteer in an experiment were found to detoxify DON to DOM-1, although the efficiency was relatively low (Gratz et al., 2013).

#### Oxidation and Epimerization

With the aid of bacteria, other detoxification processes converting DON into low-toxic products, such as oxidation of DON to 3-keto DON and epimerization of DON to 3-epi DON, have been reported (McCormick, 2013). For instance, the Gram-positive genus *Nocardioides* and the Gram-negative genus *Devosia* could achieve the detoxification processes (Ikunaga et al., 2011; Sato et al., 2012). A recent study reported that a bacterium *Devosia mutans* 17-2-E-8 could completely detoxify DON into 3-epi DON and 3-keto DON, and 3-epi DON was the major product, meanwhile the authors confirmed that 3-epi DON was much less toxic than DON by both *in vitro* and *in vivo* studies (He et al., 2015).

#### Glycosylation

Mycotoxin glycosides known as detoxification products in plants, are generally termed as masked mycotoxin, since their conjugate structures may escape routine detection by conventional analytical methods. Plants have the capacity to detoxify harmful compounds like mycotoxin by conjugation with sugars (Berthiller et al., 2007). The first UDP-glucosyltransferase (DOGT1) that can convert DON to D3G was identified from *Arabidopsis thaliana* in 2003 (Poppenberger et al., 2003). It has been verified that resistant wheat lines are capable of converting more DON to D3G, so the D3G/DON ratios of different wheat lines could give a clear indication of their resistance against DON (Cirlini et al., 2013). Results have illustrated that it is a feasible way to convert DON to D3G in transgenic cereal crops by a high-efficiency and stable UDP-glucosyltransferase to against DON contamination (Lulin et al., 2010; Schweiger et al., 2010; Shin et al., 2012; Li et al., 2015).

Generally, the detoxification enzymes can be applied after harvest to manage contaminated cereal grains, or be expressed in genetically modified crops by transgenic technologies to detoxify DON in infected grains and increase crop resistance against pathogens (He et al., 2010). It is expected that these approaches will be of great significance to reduce DON contamination in years to come.

## Conclusion and Perspectives

Functional BCAs offer alternative strategies to control DON contamination in a green and environment-friendly way. As an emerging technique, biological control including the application of beneficial organisms and their functional products such as enzymes or metabolites, has gained more and more attention in recent years (Vinale et al., 2008). In this review, we summarize different types of functional BCAs used to achieve control of DON contamination (**Figure 1B**). These control strategies mainly include prevention before harvest and detoxification after harvest. What is more, for the purpose of controlling DON contamination, it seems more effective to integrate all available BCAs flexibly throughout grain production and storage.

Researches on DON contamination control by BCAs are still developing and ongoing, since few of them are commercially available (Wegulo et al., 2015). More in-depth studies should be conducted in this field. For instance, the stability and toxicity of detoxified DON should be studied and assessed for providing food safety assurance. The detoxification mechanisms need further investigation as well. With regard to antagonistic microbes and natural fungicides, laboratory-scale studies are insufficient, so systematic field tests should be carried out to establish a comprehensive safety evaluation. In addition, with the rapid development of molecular biology and transgenic techniques, there is a need to seek and identify the genes coding effective and applicable detoxification enzymes in both microbes and plants. And detoxification genes and related enzyme products could be modified in a highly efficient, stable and safe way. With the development of emerging BCAs, there is no doubt that the application of biological control would be a promising strategy to control DON contamination in cereal grains and reduce the risk of food safety in the food chain.

## Author Contributions

AW and YT designed the work plan of this review and initiated it, and YT drafted this review with YLT, NL, CS, YL, and SW. AW and YT reviewed the final version before submission.

## Conflict of Interest Statement

The authors declare that the research was conducted in the absence of any commercial or financial relationships that could be construed as a potential conflict of interest.
